# Eastern Canadian Gastrointestinal Cancer Consensus Conference 2019

**DOI:** 10.3390/curroncol28030185

**Published:** 2021-05-26

**Authors:** Joanna Gotfrit, Rachel Goodwin, Timothy Asmis, Angela J. Hyde, Thierry Alcindor, Francine Aubin, Scott Berry, Dominick Bossé, Colin Brown, Ronald Burkes, Margot Burnell, Bruce Colwell, Jessica Corbett, Jeff Craswell, Nathalie Daaboul, Mark Doherty, D. A. Barry Fleming, Luisa Galvis, Rakesh Goel, Mohammed Harb, Alwin Jeyakumar, Derek Jonker, Erin Kennedy, Michael Lock, Aamer Mahmud, Patrick H. McCrea, Vimoj Nair, Rami Nassabein, Carolyn Nessim, Ravi Ramjeesingh, Muhammad Raza, Wissam Saliba, Satareh Samimi, Simron Singh, Stephanie Snow, Mustapha Tehfé, Michael Thirlwell, Mario Valdes, Stephen Welch, Michael Vickers

**Affiliations:** 1 The Ottawa Hospital Cancer Centre, Ottawa, ON K1H 8L6, Canada; jgotfrit@toh.ca (J.G.); rgoodwin@toh.ca (R.G.); tiasmis@toh.ca (T.A.); ahyde@mun.ca (A.J.H.); dbosse@toh.ca (D.B.); rgoel@toh.ca (R.G.); djonker@toh.ca (D.J.); vnair@toh.ca (V.N.); cnessim@toh.ca (C.N.); 2 McGill University Health Centre, Montreal, QC H4A 3J1, Canada; thierry.alcindor@mcgill.ca (T.A.); michael.thirlwell@mcgill.ca (M.T.); 3 Centre Hospitalier de l’Université de Montréal (CHUM), Montreal, QC H2X 3E4, Canada; francine.aubin.chum@ssss.gouv.qc.ca (F.A.); rami.nassabein@hotmail.com (R.N.); matohfe@gmail.com (M.T.); 4 Department of Oncology, Cancer Centre of Southeastern Ontario, Queen’s University, Kingston, ON K7L 5P9, Canada; scott.berry@kingstonhsc.ca (S.B.); aamer.mahmud@kingstonhsc.ca (A.M.); 5 PEI Cancer Treatment Centre, Charlottetown, PE C1A 8T5, Canada; cdbrown@ihis.org (C.B.); jesscorbett@hotmail.com (J.C.); patrickheathmccrea@hotmail.com (P.H.M.); 6 Mount Sinai Hospital, Toronto, ON M5G 1X5, Canada; ron.burkes@sinaihealth.ca (R.B.); erin.kennedy@sinaihealthsystem.ca (E.K.); 7 Saint John Regional Hospital, Saint John, NB E2L 4L2, Canada; margot.burnell@horizonnb.ca; 8 Queen Elizabeth II Health Sciences Centre, Halifax, NS B3H 2Y9, Canada; bruce.colwell@nshealth.ca (B.C.); alwin.jeyakumar@nshealth.ca (A.J.); ravi.ramjeesingh@nshealth.ca (R.R.); stephanie.snow@nshealth.ca (S.S.); 9 Health PEI, Charlottetown, PE C1A 6A5, Canada; jcraswell2000@yahoo.com; 10 Centre Intégré de Cancérologie de la Montérégie, Hôpital Charles LeMoyne, Longueuil, QC J4V 2G9, Canada; nathalie.daaboul@usherbrooke.ca; 11 Sunnybrook Odette Cancer Centre, Toronto, ON M4N 3M5, Canada; markdohertysunnybrook@gmail.com (M.D.); simron.singh@sunnybrook.ca (S.S.); 12 Department of Surgery, Memorial University, St. John’s, NL A1C 5S7, Canada; dabarryfleming@gmail.com; 13 Dr Everett Chalmers Regional Hospital, Fredericton, NB E3B 5N5, Canada; dr.luisa.galvis@horizonnb.ca (L.G.); drraza96@hotmail.com (M.R.); 14 The Moncton City Hospital, Moncton, NB E1C 6Z8, Canada; dr.mohammed.harb@horizonnb.ca; 15 London Regional Cancer Program, London, ON N6A 5W9, Canada; michael.lock@lhsc.on.ca (M.L.); stephen.welch@lhsc.on.ca (S.W.); 16 Cape Breton Cancer Centre, Sydney, NS B1P 1P3, Canada; wissam.saliba@nshealth.ca; 17 Hôpital Sacré-Coeur de Montréal, Montreal, QC H4J 1C5, Canada; setarehsoheil@hotmail.com; 18 Grand River Regional Cancer Centre, Kitchener, ON N2G 1G3, Canada; mario.valdes@grhosp.on.ca

**Keywords:** guidelines, anal cancer, biliary tract cancer, colon cancer, gastric cancer, rectal cancer, chemotherapy, radiation therapy, surgery

## Abstract

The annual Eastern Canadian Gastrointestinal Cancer Consensus Conference 2019 was held in Morell, Prince Edward Island, 19–21 September 2019. Experts in medical oncology, radiation oncology, and surgical oncology who are involved in the management of patients with gastrointestinal malignancies participated in presentations and discussion sessions for the purpose of developing the recommendations presented here. This consensus statement addresses multiple topics in the management of anal, colorectal, biliary tract, and gastric cancers, including: radiotherapy and systemic therapy for localized and advanced anal cancer; watch and wait strategy for the management of rectal cancer; role of testing for dihydropyrimidine dehydrogenase (DPD) deficiency prior to commencement of fluoropyrimidine therapy; radiotherapy and systemic therapy in the adjuvant and unresectable settings for biliary tract cancer; and radiotherapy and systemic therapy in the perioperative setting for early-stage gastric cancer.

## 1. Introduction

The annual Eastern Canadian Gastrointestinal Cancer Consensus Conference 2019 was held in Morell, Prince Edward Island, 19–21 September 2019. The purpose of the conference was to develop consensus statements on emerging and evolving treatment paradigms. Participants were Canadian medical oncologists, radiation oncologists, and surgical oncologists from across Ontario, Quebec, and the Atlantic provinces. Consensus statements were developed following rapid review presentations and discussion of available literature. The recommendations proposed here represent the consensus opinions of physicians involved in the care of patients with gastrointestinal malignancies who participated in this meeting.

### Basis of Recommendations

The existing scientific evidence was presented and discussed at the meeting. Recommendations were formulated within the group and categorized by level of evidence as follows:

- Level I: evidence from randomized controlled trials
- Level II-1: evidence from controlled trials without randomization
- Level II-2: evidence from analytic cohorts or case-control studies, preferably from more than one center or research group
- Level II-3: evidence from comparisons between times or places with and without the intervention
- Level III: Opinion of respected authorities based on clinical experience; descriptive

## 2. Anal Cancer

Question 1:

What is the optimal management of localized anal cancer?

- The standard treatment for localized T2-4, N0-1 anal cancer is chemoradiotherapy with mitomycin C (MMC)/5-fluorouracil (5FU). The addition of chemotherapy improves local control and reduces the need for colostomy/salvage surgery. (Level I)
- The radiation dose is 54–60 Gray (Gy) for treatment dose and 36–45 Gy for prophylactic dose. Radiotherapy planning should employ modern techniques that optimize dose delivery to minimize acute toxicities. (Level I)
- Dose escalation beyond 60 Gy increases toxicity of treatment without significant improvement in outcomes. (Level I)
- MMC may be given at a dose of 10 mg/m^2^ on d1+29 or as a single dose of 12 mg/m^2^ on d1. (Level I)
- Capecitabine may be substituted for 5FU, avoiding the need for a central venous access device and infusion pump. In case-control studies, capecitabine appears equally effective and possibly associated with less mucositis, diarrhea, and nausea but more hand-foot syndrome. (Level II/III)
- There is no role for either induction (prior to radiation) or maintenance (after radiation) chemotherapy. (Level I)
- T1N0 disease should be discussed at multidisciplinary rounds. For highly selected patients, radiation alone or local excision may be a reasonable option. Trials employing de-intensification of therapy should be supported. (Level II/III)
- Radiotherapy alone can be considered in well-selected patients not eligible for chemotherapy. (Level III)
- Patients who are HIV positive should receive the same treatment as HIV negative patients, particularly when HIV infection is well-controlled with anti-retroviral therapy. (Level III)

Evidence Summary:

Anal cancer typically refers to squamous cell carcinoma (SCC) of the anal canal. Although relatively rare, the incidence in Canada increased by an average of 3.1% from 1992–2012 with an estimated 585 new cases diagnosed in 2016 and the majority due to HPV infection (80–90%) [1]. Prognosis for anal cancer is mainly dependent on tumor size and nodal status, with lower five-year survival with advancing tumor stage and nodal positivity [2]. Historically, the treatment of anal cancer relied on surgical removal via abdominoperineal resection (APR), resulting in a permanent colostomy. Advances were made in the 1970s, when investigators at Wayne State incorporated pre-operative chemoradiation (Nigro regimen) with 5FU plus MMC, resulting in impressive pathologic complete response (pCR) rates [3]. Subsequent trials from Europe comparing chemoradiation (CRT) with radiation (RT) alone confirmed the benefits of chemotherapy with improvements in locoregional relapse and colostomy free survival (CFS) [4,5]. RTOG 87-04 and RTOG 98-11 established the combination of infusional 5-FU 1000 mg/m^2^ on days 1–4 and 29–32 plus MMC 10 mg/m^2^ on day 1 and 29 as the optimal chemotherapy concurrent with RT [6,7] for patients with T2-4, N0-1 anal cancer. With chemoradiation, at four years, it has been shown that the colostomy rate is 9% and colostomy-free survival is 71% [6]. Acceptable dosing of MMC may be either 10 mg/m^2^ on day 1 and 29 or 12 mg/m^2^ on day 1 only and due to convenience and a favorable side-effect profile, capecitabine may be substituted for 5-FU, although randomized evidence is lacking [8,9]. Studies investigating induction and maintenance chemotherapy have failed to show improvements in clinically important outcomes [10,11].

The standard dosing of radiation utilized in prospective clinical trials has ranged from 54–60 Gray (Gy) for treatment dose in 1.8 to 2 Gy per fraction and 36–45 Gy for prophylactic dose. Investigations into dose escalation showed no benefits in local control and possibly higher rates of severe acute toxicity [12,13].

Patients with T1N0 disease have been underrepresented in randomized clinical trials; however, case series suggest that this group has a favorable prognosis [14,15]. While most patients in North America receive CRT, this may be overtreatment for patients with smaller T1 disease (<1.5 cm), and multi-disciplinary discussion is recommended to determine if less intensive strategies are appropriate (such as RT alone or local excision) [16].

While some studies suggest that HIV-positive patients may experience more treatment-related toxicity, locoregional control and overall survival (OS) are similar to that of non-HIV-infected patients [17,18]. Standard CRT should be considered in this patient population with close monitoring.

Question 2:

What is the optimal management of metastatic/unresectable, recurrent anal cancer?

- Carboplatin/paclitaxel is recommended as first line therapy, with less toxicity and improved survival compared to Cisplatin/5FU. (Level I)
- Immunotherapy is active in pretreated metastatic/unresectable, recurrent anal cancer with objective response rate (ORR) 20–24%. Patients should be enrolled in studies where available. Nivolumab or pembrolizumab could be considered where available to a patient, although impact on survival is uncertain with current evidence. (Level II)

Evidence Summary:

Although most patients with localized disease are cured with definitive chemoradiation, 15–20% of patients have or develop metastatic disease at some point in their disease trajectory [4,19,20]. The most common site of metastases is the liver, and the mainstay of treatment remains systemic therapy. Select patients with oligometastatic disease should be discussed at MCC rounds for consideration of aggressive management, such as resection. Until recently, cisplatin + infusional 5FU has been the regimen of choice with institutional series reporting response rates ranging from 35–55% and median OS of 20–22 months [21,22,23]. The InterAAct trial randomized 91 patients with untreated, advanced anal SCC to carboplatin (AUC 5 D1) plus weekly paclitaxel (80 mg/m^2^ D1, D8, D15) every 28 days or cisplatin (60 mg/m^2^ D1) plus infusional FU (1000 mg/m^2^ D1–4) every 21 days [24]. Though no difference was found in response rates (59 vs. 57%) and median progression-free survival (PFS) (8.1 vs. 5.7 months), carboplatin plus paclitaxel was associated with a trend toward longer OS (20 vs. 12.3 months) and a better toxicity profile (serious adverse events 36% vs. 62%). Importantly, there was much less grade 3/4 mucositis (0 vs. 26%) and nausea (2 vs. 17%) with carboplatin plus paclitaxel. As a result, carboplatin plus paclitaxel is now the preferred option for patients with untreated, advanced anal SCC.

Since many anal SCCs express PD-L1 (56%) and > 70% are associated with HPV infection (leading to increased tumor-infiltrating lymphocytes), there is great interest in immunotherapy strategies [25,26,27]. In the refractory setting, both nivolumab and pembrolizumab have been evaluated in single arm, phase II trials [28,29]. The response rate was 24% for nivolumab and 17% for pembrolizumab, with both PD-1 inhibitors producing a tolerable toxicity profile. Clearly, these agents have activity in anal SCC but have not been compared to best supportive care (or each other), and therefore definitive conclusions about benefits in survival cannot be ascertained at present.

Question 3:

What is the optimal follow-up schedule for a patient with locally advanced anal cancer who has completed chemoradiotherapy?

- First clinical assessment including DRE and inguinal node palpation should be between six to eight weeks post-therapy. Close observation every one to three months can be maintained up to 26 weeks post-treatment or until complete response. Assessment every three to six months should be undertaken up to three years. (Level III)
- Since a subset of patients will respond more slowly, in the absence of progression, treatment failure should not be declared before six months post-treatment. (Level I)
- Routine biopsies should not be considered unless there is a strong concern of persistent or progressive disease. (Level III)
- If biopsies confirm persistent disease after 26 weeks post-therapy or progressive disease at any time, the patient should have restaging investigations and be considered for salvage surgery. (Level II-2)

Evidence Summary:

There is a lack of prospective trials evaluating the optimal surveillance strategy for treated anal cancer patients. The recommendations for follow-up are largely based on the treatment and assessment schedule from the ACT-II study [30] and NCCN guidelines [31]; without availability of high-level comparative evidence, clinical discretion may be appropriate. Anal SCCs are known to regress slowly and may continue to respond up to 26 weeks post-CRT, and therefore treatment failure should not be determined before this timeframe [32]. As well, since the majority of recurrences develop within the first three years post-therapy, it would be advisable to continue surveillance at least until this timepoint [30].

## 3. Non-Operative Management of Rectal Cancer

Question 1:

Following neoadjuvant chemoradiotherapy for localized rectal cancer, some patients will have a clinical complete response and may never recur. What is the role of non-operative management and a wait and watch approach for these patients?

- Surgery after neoadjuvant treatment remains the standard of care in locally advanced rectal cancer (stage II and III), but watch and wait may be offered to patients who achieve a clinical complete response, ideally in a clinical trial context or when requested by informed patients. This can be considered at institutions with adequate expertise in the surveillance associated with this approach. (Level III)
- The first reassessment should be done no sooner than 8 weeks after treatment, and waiting up to 14–16 weeks may also be reasonable. Surveillance may include DRE, endoscopy, MRI, CT scans, and CEA up to every three months for the first year, with declining frequency over five years. Biopsies should be done if residual disease/recurrence is suspected. (Level III)

Evidence Summary:

The non-operative management of rectal cancer is emerging as a potential new standard of care for patients who achieve a complete clinical response following neoadjuvant chemoradiotherapy. The evidence to support this strategy comes largely from expert opinion, evidence from multiple systematic reviews, and from the International Registry, which do show favourable outcomes for both disease-free survival (DFS) and OS [33,34,35]. Several authors have recently published criteria for a complete clinical response and regression schemas [36,37,38,39]. Studies suggest various protocols for watchful waiting, encompassing varying frequencies of surveillance imaging, and other investigations [40,41,42]. A large phase II study is ongoing at the time of this conference and may further clarify the optimal treatment for patients [43]. If undertaken, non-operative management should be done at high-volume centres dedicated to the assessment and follow-up of these patients.

Question 2:

Should chemotherapy (induction/consolidation) be a routine part of the W&W approach?

- There is currently a paucity of definitive evidence on the role of chemotherapy in the induction and consolidation settings. (Level III)

Evidence Summary:

While there is a lack of definitive evidence on the role of chemotherapy in the watch and wait approach in rectal cancer, a phase II non-randomized trial evaluating mFOLFOX6 after neoadjuvant chemoradiation (before surgery) in locally advanced rectal cancer showed that chemotherapy had the potential to increase the rates of pathologic complete response and may have the potential to increase the proportion of patients eligible for less invasive strategies [44]. Further studies including randomized phase III trials are needed before any definitive conclusions can be drawn or recommendations made.

Question 3:

How should near-complete clinical response be treated?

- In patients with a near-complete clinical response, the current standard of care is total mesorectal excision (TME). (Level I)

Evidence Summary:

A near-complete clinical response is defined as follows: on DRE with smooth induration or minor mucosal abnormalities; on endoscopy as irregular mucosa, small mucosal nodules, minor mucosal abnormalities, superficial ulceration, or mild persisting erythema of the scar; on MRI T2 as mostly dark T2 signal but with some remaining intermediate signal, and/or partial regression of lymph nodes; and on MR-DW as significant regression of signal [39].

Patients who do not achieve a complete clinical response should be discussed at multidisciplinary tumour board rounds and should undergo TME [39].

## 4. Dihydropyrimidine Dehydrogenase Deficiency

Question 1:

Should pre-emptive testing for dihydropyrimidine deshydrogenase (DPD) deficiency be done to improve patient safety with fluoropyrimidines?

- Patients must be informed of the DPD deficiency-associated risks with fluoropyrimidines-based therapies and the available tests to detect it. (Level III)
- Resources should be made available to provide pre-emptive *DPYD* genotyping before starting treatment with fluoropyrimydines to detect these four clinically relevant variants: DPYD*2A, c.2846A > T, c.1679T > G, and c.1236G > A. (Level II-2)
- Initial 5FU or capecitabine dosage adjustment should be done according to published guidelines for each specific *DPYD* genotype and patients’ individual characteristics and circumstances. The dose should be readjusted during subsequent treatment cycles according to patients’ tolerance in order to achieve safe maximum exposure and to optimize treatment effectiveness. (Level II-3)

Evidence Summary:

Fluoropyrimidines are commonly prescribed in the adjuvant and palliative settings of various solid malignancies including gastrointestinal, breast, head and neck, and other cancers. It is usually well-tolerated. However, severe and potentially life-threatening toxicity can occur in 5–10% of the treated population, leading to treatment discontinuation and hospitalization, with a mortality rate of 0.5–1% of treated patients [45,46]. DPD is the main metabolic enzyme necessary for fluoropyrimidine inactivation. Toxicity from fluoropyrimidines is largely associated with DPD deficiency, the most recognized cause of which is genetic polymorphisms in the *DPYD* gene [47].

There are over 100 mutations and variant alleles reported to occur in the *DPYD* gene that have mixed effects on enzyme activity. The most clinically relevant polymorphisms associated with severe toxicity include DPYD*2A, c.1679T > G, c.2846A > T, and c.1236G > A, which decrease DPD enzymatic activity by 50%, 68%, 31%, and 35%, respectively [48,49,50,51,52,53,54,55,56]. These can lead to severe and prolonged neutropenia, mucositis, and diarrhea.

In a meta-analysis of 15 studies (10 of which were prospective) between 2004 and 2011, DPYD*2A carrier status (1.46% of patients) was strongly associated with grade 3+ toxicity following fluoropyrimidine treatment (OR 5.42, 95% CI 2.79–10.52, *p* < 0.001) [48]. Specifically, DPYD*2A carriers had increased risk of grade 3+ hematologic toxicity (OR 15.77, 95% CI 6.36–39.06, *p* < 0.001), diarrhea (OR 5.54, 95% CI 2.31–13.29, *p* < 0.001), and mucositis (OR 7.48, 95% CI 3.03–18.47, *p* < 0.001). Similar associations with toxicity have been found among other *DPYD* gene variants, including the c.1679T > G variant allele [51] and the c.2846A > T [48,51] and c.1236G > A alleles [51,57].

Among 2038 patients prospectively screened for DPYD*2A gene polymorphism, 1.1% were found to be heterozygotes, and 0.1% were homozygotes. This group was treated with genotype-guided dosing of fluoropyrimidines with a median dose-intensity of 48%. When compared to historical controls, the group treated by genotype-guided dosing experienced significantly less grade 3 and 4 toxicity (28% vs. 73%, *p* < 0.001) and drug-induced death (0% vs. 10%). They experienced toxicity rates similar to wild-type patients treated with standard dosing, as well as similar active drug exposure [50]. Similarly, a prospective study investigating the impact of screening for the four most relevant *DPYD* variants (DPYD*2A, c.2846A > T, c.1679T > G, and c.1236G > A) with genotype-guided dosing showed similar results. Patients with heterozygous variants in the c.2846A > T (1.5%) and c.1236G > A (4.6%) alleles received an initial 25% dose reduction, and those with heterozygous variants in the DPYD*2A (1.5%) and c.1679T > G (0.1%) alleles received an initial 50% dose reduction. Patients with wild-type *DPYD* genes received standard dosing. Relative dose intensity among *DPYD*-variant allele carriers ranged from 53% to 74%. Overall, grade 3+ toxicity was only mildly worse in the *DPYD*-variant allele carriers (39% vs 23%, *p* = 0.0013), and there was no difference in grade 4+ toxicity (5% vs. 3%, *p* = 0.49) nor in the death rate (1% vs. <1%, *p* = 0.55) [52].

One must remember that negative genotyping does not definitively rule out DPD deficiency, and severe toxicities may still occur. This may be due to other gene polymorphisms, for example, the *TYMS* gene variation [58].

While the cost and resource requirements must be considered by institutions planning to implement DPD deficiency testing, there is some data to suggest there may be a net-cost savings from this approach [50].

## 5. Biliary Tract Cancer

Question 1:

Is there a role for radiotherapy in inoperable/unresectable patients?

- All patients with localized (resectable, unresectable, borderline resectable) cholangiocarcinoma should be discussed at multidisciplinary tumour board rounds. (Level III)
- The role of radiation therapy may be considered in unresectable cases for local control and palliative goals. Survival advantage is seen in epidemiological and retrospective studies. (Level II-2)
- SBRT is preferred for unresectable cases. Conventional radiation with higher fractionations is also an option. (Level III)
- Active trials are available and patients should be treated under protocol if possible. (Level III)

Evidence Summary:

Radiation in unresectable biliary cancer may be beneficial for local control and palliation. In a phase II study of patients with unresectable, localized intrahepatic cholangiocarcinoma who received 15 fractions of high-dose proton beam therapy (67.5 Gy peripheral, 58 Gy central, dose de-escalated based on Veff of uninvolved liver), at two years, 20% were alive without progression, 53.8% had distant metastases, 12.8% had isolated local failure, and 2.8% had local and distant failure [59]. While case series and epidemiological studies suggest a possible OS advantage, this has not been shown in high-level studies. In a retrospective study of 84 heterogeneous, unresectable, intrahepatic cholangiocarcinoma patients not treated with chemotherapy, 35 patients received radiation (30–60 Gy in 1.8–2.0 Gy per fraction), and 49 did not. The overall response rate in the primary tumour among patients receiving radiation was 37%, with 8.6% achieving complete response and 28.5% achieving partial response. In the radiation group compared to those not receiving radiation, one-year OS was 38.5% vs. 16.4%, two-year OS was 9.6% vs. 4.9%, and median survival was 9.5 vs. 5.1 months. Similarly, using the Surveillance Epidemiology and End Results (SEER) database, a retrospective review of intrahepatic cholangiocarcinomas demonstrated that OS was significantly better among patients who received radiation alone compared to no treatment (median survival seven vs. three months) [60].

Chemoradiation has not shown benefit beyond chemotherapy alone, so other approaches are preferred. In a phase II study in hilar or extrahepatic locally advanced biliary tract cancer, which was closed before completion due to slow recruitment, patients were randomized to chemotherapy with gemcitabine and oxaliplatin (GEMOX) compared to chemoradiotherapy using 5-FU and cisplatin with radiation at 50 Gy. PFS in the chemotherapy group was 11 months compared to 5.8 months in the chemoradiotherapy group, and OS was 19.9 months in the chemotherapy group compared to 13.5 months in the chemoradiotherapy group, indicating that chemotherapy alone was at least as efficient as chemoradiotherapy [61].

There appears to be dose-response relationship on local control and OS using SBRT in intrahepatic and extrahepatic cholangiocarcinoma, with higher doses improving outcomes [62]. This is supported by a phase 1 study, where ten patients with intrahepatic cholangiocarcinoma who received six fractions of radiobiologically guided stereotactic body radiation to 5%, 10%, and 20% toxicity risk (median dose 36 Gy, range 24–54 Gy) experienced a median OS of 15 months, and a one-year OS of 58% [63].

Intraluminal brachytherapy is another radiation technique with some positive results in this setting. It has the advantage of delivering a high radiation dose to the tumor volume and bypassing the radiosensitive skin and surrounding structures. Retrospective studies of intraluminal brachytherapy have demonstrated a median OS for unresectable hilar cholangiocarcinoma of 11 months [64] and almost 15 months with combined intraluminal brachytherapy and SBRT [65].

Ongoing studies investigating radiation in the locally advanced unresectable setting include NRG GI 001, a study examining OS among patients with localized unresectable intrahepatic cholangiocarcinoma who have not progressed after six months of chemotherapy (cisplatin and gemcitabine). These patients are randomized to radiation (from 37.5 Gy to 67.5 Gy) versus observation, and we eagerly await results.

Question 2:

What is the role of radiotherapy in the pre-operative or pre-transplant patient?

- Patients considered for pre-transplant radiotherapy should be treated in the context of a clinical trial. (Level III)
- Patients with borderline resectable disease may benefit from preop radiotherapy or chemoradiotherapy. (Level II-3)

Evidence Summary:

Among patients with local tumours who are considered unresectable at diagnosis, chemoradiation can be considered as neoadjuvant treatment to downstage the tumour before resection. A delay to surgery for preoperative treatment may allow an assessment of disease biology and facilitate better patient selection by limiting resections among patients with a high risk of locoregional or distant failure; these patients would avoid a futile surgery and the subsequent chance of complications and prolonged recovery. Additional rationale for preoperative treatment relates to facilitation of downstaging (converting unresectable or boderline patients to surgical or transplantable candidates), reduced margin positivity, and reduced tumour seeding. Only a few studies have examined neoadjuvant chemoradiation before resection. In a small prospective study in 1997, it was shown that neoadjuvant chemoradiation with infusional 5-FU concurrent with 45–50.4 Gy of radiation in patients with both hilar and distal cholangiocarcinoma resulted in an R0 resection in 100% (nine patients) undergoing the treatment, despite six of them being initially categorized as unresectable, compared to 54% R0 resection rate in the group receiving surgery alone [66]. We suggest strict adherence to the Mayo protocol selection criteria if this approach is undertaken (exclusion criteria: intra or extra-hepatic metastases including nodes, operative biopsy or attempted resection, active infections, previous malignancy) [67].

Beyond surgical resection, liver transplantation is another approach to consider in the setting of cholangiocarcinoma. The Mayo clinic protocol for liver transplantation in the setting of cholangiocarcinoma has reasonable survival rates. The protocol includes strict patient-selection criteria as outlined above [67]. When patients are deemed candidates, the treatment protocol includes sequential external beam radiotherapy with 5-FU chemosensitization, brachytherapy, abdominal exploration for staging, and, finally, liver transplantation. The chemoradiation regimen includes radiation to 45 Gy in 30 fractions (1.5 Gy twice daily) with concurrent infusional 5-FU 500 mg/m^2^ daily over the first three days of radiation. The brachytherapy two weeks later includes iridium-192 at a dose of 20–30 Gy. Patients then take oral capecitabine until transplant.

In the Mayo experience, among patients undergoing the transplant protocol, there was a complete pathologic response in 42% of explanted livers, with a five-year OS of 82% among patients ultimately transplanted, in comparison to 21% in those who underwent resection. Similar results were obtained by other centres in the United States [68,69]. In patients who undergo liver transplantation for hilar cholangiocarcinoma, the three-year survival rate is 40% [70], and the five-year survival rate is 20% [71].

Question 3:

What is the current role of radiotherapy in post-operative management patients with biliary tract cancer?

- Post-op chemoradiotherapy may reduce local and distant failure and improve OS based on a meta-analysis of phase II and retrospective data. (Level II)
- For resected positive margin (R1) or resected gross residual disease (R2) or positive regional nodes, chemoradiation should be considered. (Level II)
- For adjuvant radiation, the radiation target volume should include draining regional lymph nodes to 45 Gy in 25 fractions with the tumour bed receiving 50–60 Gy in 1.8 Gy to 2 Gy per fraction. (Level II)

Evidence Summary:

Local failure rates after surgery and chemotherapy remain high in the setting of cholangiocarcinoma. About 50% of patients undergoing curative resections will suffer a local recurrence [72,73]. The addition of radiation may offer a reduction in the high local failure rates; however, much of the data in this area is early phase or retrospective and must be interpreted with caution. For example, among 168 patients with fully resected extrahepatic biliary tract cancer assessed retrospectively, the group who received adjuvant chemoradiation (115 patients) experienced improved locoregional control, DFS, and OS compared to the group who received no chemoradiation, and adjuvant chemoradiation was a significant independent prognostic factor for improved outcomes in locoregional control and survival [74]. Similarly, using the Surveillance Epidemiology and End Results (SEER) database, a retrospective review of intrahepatic cholangiocarcinomas demonstrated that OS was significantly better among patients who received surgery and adjuvant radiation compared to surgery alone (HR 0.40, 95% CI 0.34–0.47) [60]. At Johns Hopkins, among 34 patients with extrahepatic or distal cholangiocarcinoma who underwent pancraticoduodenectomy followed by adjuvant 5-FU and EBRT, median survival was improved to 37 months compared to 22 months in historical controls not treated with radiation, and five-year survival was 35% [75]. In another series, among 96 patients with intrahepatic cholangiocarcinoma treated surgically (41% curative resection, 14% non-curative resection, 45% palliative stenting), patients who received post-op radiation had a one-year survival of 66% compared to 27% among those who did not receive radiation, but there was no difference in longer term survival [76]. Lastly, among 112 patients with perihilar or Klatskin tumours who underwent surgical resection, median survival was 19 months among those who subsequently underwent post-operative radiation compared to 8.3 months among those who did not [77].

While there may be a benefit to EBRT, there is less evidence for brachytherapy in this setting. A series in Amsterdam showed positive results for EBRT but with no benefit from the addition of brachytherapy [78].

In contrast to the results of retrospective studies, prospective work at Johns Hopkins demonstrated no benefit to EBRT with or without brachytherapy in the treatment of 50 patients who underwent curative or palliative surgery [79]. That said, in the prospective single arm phase II study SWOG S0809, examining patients with resected extrahepatic or gallbladder cholangiocarcinoma (pT2-4 or N+ or margin positive) treated with adjuvant gemcitabine/capecitabine for four cycles followed by chemoradiation with concurrent capecitabine (45 Gy to regional lymphatics, 54–59.4 Gy to tumour bed), there was a two-year survival of 65% and median OS of 35 months (R0, 34 months; R1, 35 months). This is significantly higher than expected based on historical controls. Similarly, the local failure rate was 30–50% better than expected, and the poorer outcome of R1 resection seemed to be eliminated by radiation [80].

A meta-analysis attempted to answer questions around the utility of radiation in the post-operative setting. In a meta-analysis examining 20 studies with 6712 patients, there was a trend toward a survival benefit for adjuvant radiation (*p* = 0.06), which was more strongly significant among the subgroups with node-positive disease or margin positivity. Those receiving adjuvant chemotherapy or chemoradiotherapy derived more benefit than adjuvant radiation alone [81].

Question 4:

What is the current role of systemic therapy in the post-operative management of patients with biliary tract cancer?

- Patients with resected gallbladder cancer or cholangiocarcinoma should be considered for adjuvant chemotherapy. Capecitabine for six months is the current regimen of choice based on randomized trial results (Level I). Risk features such as lymph node involvement and positive resection margin should be considered in decision-making.
- Surveillance with no adjuvant therapy is an option, particularly in early-stage, node-negative disease with clear margins. (Level II-1)
- Patients with resected periampullary tumors should be considered for adjuvant gemcitabine chemotherapy. (Level I)
- Current evidence suggests gemcitabine alone or combined with oxaliplatin is not associated with benefit in the adjuvant setting for cholangiocarcinoma or gallbladder cancer (Level I). Other regimens (including gemcitabine/cisplatin) are currently under investigation

Evidence Summary:

Surgery is the only curative treatment for biliary tract cancer; however, surgical outcomes are poor. Multiple studies have examined the impact of adjuvant chemotherapy in this setting. The phase III randomized BILCAP trial [82], which included intrahepatic, extrahepatic, and gallbladder cancers (but not ampullary), showed that six months of adjuvant capecitabine (1250 mg/m^2^ po BID) significantly improved OS (51 vs. 36 months) when the results were analyzed per protocol and adjusted for prognostic factors, including nodal status, grade, and gender. In subgroup analyses, the results remained positive for both lymph-node-positive and -negative disease and for R0 and R1 resections.

The phase III randomized ESPAC-03 trial [83], which included a large majority of ampullary cancers, demonstrated that adjuvant treatment with neither 5-FU nor gemcitabine improved OS compared to no adjuvant chemotherapy, although after adjusting for prognostic variables (age, bile duct cancer, poor tumour differentiation, positive lymph nodes) there appeared to be a survival benefit for adjuvant chemotherapy (HR 0.75, *p* = 0.03), particularly gemcitabine (HR 0.70, *p* = 0.03). As such, it is reasonable to consider adjuvant gemcitabine in the adjuvant treatment of periampullary tumours.

Outside of peri-ampullary cancers, there is no evidence for adjuvant gemcitabine-based chemotherapy. The phase III randomized PRODIGE 12 trial [84], which included all cholangiocarcinomas and gallbladder cancers but excluded ampullary cancers, showed that adjuvant chemotherapy with gemcitabine and oxaliplatin did not improve relapse-free survival. It is important to note that this study included a large majority of node-negative tumours with R0 resections, which may have had a better baseline prognosis. On the same note, the phase III randomized BCAT trial [85], which included perihilar and distal extrahepatic cholangiocarcinomas (but not gallbladder or ampullary cancers), showed that adjuvant gemcitabine had no benefit on OS but again included a large majority of node-negative tumours with R0 resections.

## 6. Gastric Cancer

For this consensus, we focused our evidence on non-cardia gastric cancer (site of initiation > 2 cm from the gastroesophageal (GE) junction). It is important to recognize that the majority of gastric cancer trials enrolled patient with clinical stage T2N0 disease or higher (at least invasion beyond the submucosa). This consensus does not outline the treatment of T1N0 disease. 

Question 1:

What are the systemic therapy options available to improve outcomes in patients with early gastric cancer?

- Perioperative FLOT (docetaxel, oxaliplatin, and leucovorin (LV) with short term 24-h infusional 5-FU) chemotherapy is the preferred approach as it produces the highest survival rate in regimens tested in phase III trials, but should be reserved to fit patients. (Level I)
- Consideration can be given to CF or FOLFOX in the perioperative setting for patients requiring a less intensive regimen than FLOT based on expert opinion. (Level II-1)

Evidence Summary:

Perioperative FLOT chemotherapy is the preferred approach for resectable gastric cancer among patients with good performance status. The phase II/III FLOT4-AIO trial enrolled patients with clinically staged T2N0 or higher disease and compared perioperative FLOT to perioperative ECX or ECF. This trial enrolled 300 patients in the phase II component and 716 in the phase III component (44% gastric and 56% gastroesophageal junction). The primary endpoint of this trial was positive with significantly improved median OS (50 vs. 35 months, HR 0.77, 95% CI 0.63–0.94) and three-year OS (57% versus 48%) [86]. The rates of perioperative complications were not higher in the FLOT group (50%) versus the ECF/ECX group (51%). As expected, there were slightly more grade three and four toxicities with FLOT including diarrhea, neutropenia, infections, and sensory neuropathy.

If there are concerns regarding the use of FLOT (performance status, co-morbidities, organ function), then we recommend consideration be given to CF or FOLFOX in the perioperative setting. This recommendation is based on the pivotal MAGIC (United Kingdom Medical Research Council Adjuvant Gastric Infusional Chemotherapy) trial. This trial randomized patients to three cycles of epirubicin, cisplatin, and 5-FU (ECF) both before and after surgery versus surgery alone and showed improved OS in the chemotherapy arm (five-year OS 23% vs. 36%, HR for death 0.75, 95% CI 0.60–0.93) [87]. Additional studies and expert opinion have supported omitting epirubicin without compromising the efficacy of chemotherapy [88]. In addition, if the patient is unable to tolerate FLOT, eliminating the docetaxel and giving FOLFOX is an acceptable standard [89,90,91].

Question 2:

What is the role of radiotherapy in resected early-stage gastric cancer patients who received pre-operative chemotherapy?

- The role of radiotherapy in the curative management of resectable, early-stage gastric cancer who received peri-operative FLOT/ECF chemotherapy is not clear. (Level I)
- Routine postoperative chemoradiation is not indicated, but after multidisciplinary discussion, postoperative chemoradiation therapy could be considered in resected gastric cancer at high risk for relapse with the aim to improve local recurrence (e.g., R1 or R2 resection). (Level III)

Evidence Summary:

The role of radiotherapy in the curative management of resectable, early-stage gastric cancer who received peri-operative FLOT is not clear and has not been tested. Given our preferred peri-operative regimen is FLOT chemotherapy and that the sentinel trial did not use radiation [86], routine postoperative radiation is not indicated.

In the Dutch CRITICS randomized trial [92], patients with stage IB to IV potentially resectable gastric cancer received induction chemotherapy with epirubicin, cisplatin/oxaliplatin, and capecitabine (ECX or EOX). After surgery, which required at minimum a D1 lymphadenectomy, patients were treated with either three cycles of the same chemotherapy regimen or chemoradiotherapy (45 G in 25 fractions with weekly cisplatin and daily capecitabine). There was no significant improvement in OS, PFS, or local recurrence rates. A meta-analysis of six trials, including the CRITICS trial, concluded there was a trend toward a survival benefit of adjuvant chemoradiotherapy compared to adjuvant chemotherapy, although this was not statistically significant [93]. Therefore, routinely replacing the post-operative chemotherapy with radiation is not recommended. However, after multidisciplinary discussion, postoperative chemoradiation could be considered in resected gastric cancer patients who are at high risk of relapse with the aim to improve local recurrence rates (example: R1 or R2 resection).

Question 3:

What is the role of adjuvant treatment in patients with early-stage gastric cancer who underwent upfront resection?

- The preferred approach is perioperative treatment, but if the patient has upfront surgical resection, then there are two options available for discussion in post-operative management:
  - Adjuvant chemo-radiotherapy (based on the adequacy of D2 resection). (Contradicting Level I evidence for and against this option)
  - Adjuvant chemotherapy with the CAPOX regimen for six months. (Level I)

Evidence Summary:

Although not the preferred approach, for patients who have completed potentially curative gastric surgery without neoadjuvant therapy, there are two options available and supported by clinical trials for post-operative management: chemotherapy or chemoradiation. For patients who have not undergone an adequate D2 resection (at least 16 lymph nodes in total) then strong consideration of chemoradiation is warranted. This recommendation is based on the MacDonald trial [94] that showed an OS benefit in the adjuvant chemoradiotherapy group compared to surgery alone, in a setting where only 10% of patients had a D2 dissection. The three-year and five-year OS rates were 50% vs. 41% and 48% vs. 31%, respectively. Criticisms of this trial include lack of D2 dissection, outdated radiation techniques, and an outdated and toxic bolus 5-FU regimen. More often now when giving MacDonald protocol, oncologists recommend less toxic infusional 5-FU (200 mg/m^2^ per day for the duration of radiation) or concurrent oral capecitabine (825 or 850 mg/m^2^ twice daily).

If adjuvant chemotherapy is given alone, we recommend the use of CAPOX based on the CLASSIC trial [95]. This trial enrolled 1035 patients from South Korea, China, and Taiwan with stage II, IIIA, or IIIB gastric cancer and randomly assigned them to eight cycles of chemotherapy versus surgery alone after D2 gastrectomy. Despite the fact that only 67% of the patients assigned to chemotherapy received all eight cycles, chemotherapy improved the three-year disease-free survival (74% versus 59%, HR for death 0.56, 95% CI 0.44–0.72). At five years, the OS advantage was statistically significant (78% versus 69%, HR for death 0.66, 95% CI 0.51–0.85).

Question 4:

What is the role of pre-operative radiotherapy in resectable or borderline resectable, early gastric cancers?

- The role of neoadjuvant radiation therapy or chemoradiation therapy for borderline resectable, true gastric cancers is unclear and should not be routinely done. Consider only treating on a clinical trial. (Level II-3)

Evidence Summary:

There is a paucity of data on preoperative radiotherapy in resectable or borderline resectable, early gastric cancer. The POET trial [96] investigated preoperative chemotherapy versus chemoradiotherapy in locally advanced adenocarcinoma of the gastroesophageal junction, and although there was a trend toward improved three-year OS in the chemoradiation group, it was not statistically significant. Freedom from local tumour progression was significantly increased. The ongoing TOPGEAR trial will help clarify the potential role of preoperative radiation.

Question 5:

In the case of non-response to the preoperative part of the perioperative regimen, what is the best approach?

- The prognosis of these patients with poor metabolic or pathologic response is guarded whether the same regimen is continued or replaced by chemoradiotherapy. (Level II)
- It is unknown whether a switch to a non-cross-resistant regimen would improve the prognosis. A clinical trial, if available, would be the best option for these patients. (Level III)

Evidence Summary:

Among patients with a poor response to pre-operative chemotherapy, the best subsequent management is unclear. It is reasonable to conclude if pre-operative chemotherapy had minimal effect on the tumour, more of the same chemotherapy is unlikely to provide benefit. There is a lack of high-level data assessing whether outcomes are improved by switching to a different non-cross resistant chemotherapy. Therefore, management of these patients needs to be individualized and discussed at multi-disciplinary case rounds. We would advocate for a clinical trial addressing this topic.

Question 6:

Is there a role for biologics, targeted therapy, or immunotherapy in early-stage gastric cancer?

- At present there is no evidence to support the use of biologics, targeted therapy, or immunotherapy in early-stage gastric cancer.
- Testing for microsatellite instability (MSI) status should be considered to help guide treatment decisions and discussions around the possibility of lack of benefit of chemotherapy in MSI-high tumours. (Level II)

Evidence Summary:

Data has emerged regarding MSI as a prognostic and predictive biomarker for chemotherapy in the curative gastric cancer setting. MSI-high gastric cancers are found in approximately 5% to 10% of resectable gastric cancers, and patients are more often characterized as female, older age, intestinal histological type, mid/lower gastric location, absence of lymph node metastases, and stage I-II [97]. The MAGIC trial, a randomized-controlled trial of resectable patients, was the first to publish the correlation of improved OS among patients with MSI-high tumours in comparison to those whose tumours were microsatellite stable (MSS) or MSI-low in the group who received surgery alone (HR 0.35; 95% CI 0.11–1.11, *p* = 0.8) [98]. In contrast, patients in the perioperative chemotherapy arm of the MAGIC trial had worse outcomes in the MSI-high group (HR 2.22; 95% CI 1.02–4.85 *p* = 0.04). The CLASSIC trial also showed similar results where patients with MSI-high, resectable gastric cancer gained no survival benefit from adjuvant chemotherapy compared to surgery alone: the five-year disease-free survival for MSI-H patients in the adjuvant chemotherapy arm was 83.9% versus 85.7% in the surgery alone group [99]. Pietrantonio et al. [100] performed individual patient data meta-analysis (n= 1556) on the prognostic/predictive effect of MSI in patients with resectable gastric cancer enrolled in four clinical trials (MAGIC, CLASSIC, ARTIST, and ITACA-S). Once again, this confirmed the predictive value of MSI status in resected gastric cancer. Patients with MSS/MSI-low gastric cancers benefitted from the addition of chemotherapy whereas those with MSI-high cancers did not benefit. The five-year OS among patients with MSI-high gastric cancer who received chemotherapy with surgery was 75% versus 83% in those treated with surgery alone (HR 1.50; 95% CI, 0.55–4.12). Due to this evidence, we encourage testing and identification of MSI-high gastric cancer patients and to discuss the potential lack of benefit from chemotherapy in the curative setting. The effect of MSI on taxane-containing perioperative chemotherapy regimens needs to be investigated.

## Data Availability

Not applicable.
